# Application of an Eco-Friendly Antifungal Active Package to Extend the Shelf Life of Fresh Red Raspberry (*Rubus idaeus* L. cv. ‘Kweli’)

**DOI:** 10.3390/foods11121805

**Published:** 2022-06-19

**Authors:** Tiago M. Vieira, Vítor D. Alves, Margarida Moldão Martins

**Affiliations:** LEAF—Linking Landscape, Environment, Agriculture and Food, Associated Laboratory TERRA, Instituto Superior de Agronomia, Universidade de Lisboa, Tapada da Ajuda, 1349-017 Lisbon, Portugal; tdmvieira@gmail.com (T.M.V.); mmoldao@isa.ulisboa.pt (M.M.M.)

**Keywords:** antifungal active packaging, raspberry, chitosan, green tea extract, rosemary extract

## Abstract

The main objective of this study was to extend the shelf life of fresh red raspberry (*Rubus idaeus*. L. cv. ‘Kweli’) by using active film-pads inside commercial compostable packages. The pads were produced with chitosan (Ch) with the incorporation of green tea (GTE) and rosemary (RSME) ethanolic extracts as natural antifungal agents. Pads were placed on the bottom of commercial fruit trays underneath the fruits, and the trays were heat-sealed with a polyacid lactic (PLA) film. Preservation studies were carried out over 14 days of storage at refrigeration temperature (4 °C). Raspberry samples were periodically analyzed throughout storage, in terms of quality attributes (fungal decay, weight loss, firmness, surface color, pH, total soluble solids), total phenolic content and antioxidant activity. Gas composition inside the packages was also analyzed over time. From the packaging systems tested, the ones with active film-pads Ch + GTE and Ch + RSME were highly effective in reducing fungal growth and decay of raspberry during storage, showing only around 13% and 5% of spoiled fruits after 14 days, respectively, in contrast with the packages without pads (around 80% of spoiled fruits detected). In addition, fruits preserved using packages with Ch + RSME active film-pads showed lower mass loss (5.6%), decreased firmness (3.7%) and reduced antioxidant activity (around 9% and 15% for DPPH and FRAP methods, respectively). This sustainable packaging presents a potential strategy for the preservation of raspberries and other highly perishable small fruits.

## 1. Introduction

Raspberry commercialization has recently undergone significant changes due to increasing customer quality requirements, health and lifestyle (sustainable consumption) concerns, and the demand throughout the year [1]. Such changes require producers and traders’ access to new varieties and to develop new strategies to reduce softening of the fruits and improve shelf life. Innovation in packaging technology can play a key role in improving the post-harvest quality. Because raspberries provide an important source of nutraceutical compounds (high phenolic compounds and anthocyanin contents) that are beneficial for human health [2], their marketing must integrate the themes of sustainable production and distribution processes [1,3]. The supply chain must consider packaging management, and the use of biodegradable materials derived from renewable sources is one key factor to increase sustainability of the post-harvest supply chain.

Raspberry fruits stored at 0–0.5 °C and 90–95% relative humidity (RH) can be maintained in an ambient atmosphere for 5–7 days. High CO_2_ treatments and modified atmospheres (15–20% CO_2_ and 5–10% O_2_) (MAP) have also been studied [1] for improving the berries’ shelf life. MAP is an active or passive dynamic process that involves the use of plastic films that limit gas diffusion from inside to outside and vice versa, leading to CO_2_ enrichment and O_2_ reduction inside the package [4]. Active MAP results from a rapid process of gas replacement establishing the desired gas mixture inside a package. On the other hand, passive MAP is generated by the natural process of fruit respiration and film permeability, and the gas composition eventually reaches a steady-state value, which should be suitable for the packed product [5]. The gaseous composition at steady-state depends on a series of factors, such as mass of the packed product, storage temperature, fruit respiration rate, cultivar and ripening stage. Moreover, the exchange of gases between the atmosphere inside the package and the exterior is affected by gas concentration differences, the exposed surface and permeability of the selected film [1]. Several films with various permeability values for water vapor, CO_2_ and O_2_ for fruit and vegetable packaging are commercially available [1,6], but the gas permeability values of most plastic films are too low to allow gas exchange and permit slow respiration [7]. During shipping, handling or retail display, a passive MAP storage system can cause O_2_ depletion and CO_2_ accumulation to levels outside the optimal range for the product, due to an inadequate permeability of the package film [8]. Raspberries should not be exposed to CO_2_ levels greater than 20%, which can cause discoloration, softening and the development of off-flavors [1].

In the soft fruit sector, packaging is changing, and the reduction in packaging weight and the use of sustainable materials is becoming essential to respond to the increased environmental concerns of retailers and customers. Alternative biodegradable and sustainable packaging materials to the traditional non-biodegradable oil-based polymers are starting to be used in packaging for fresh and fresh-cut produce [1]. Polyester poly (lactic acid) (PLA) is one of the most economically competitive alternatives to traditional oil-based polymers, such as polyethylene terephthalate (PET) and high- and low-density polyethylene [9,10]. It can be produced from renewable sources, such as sugar beet and corn starch, or other renewable biomass products and wastes [11]. Several studies have shown the potential for this alternative material to be used for post-harvest storage of fruits [9,12,13], but data on the use of these materials to store highly perishable raspberry fruits under passive MAP conditions are limited [1].

Active packaging is another innovative approach that has emerged to maintain quality and extend the shelf life of fruits [14]. This packaging system is designed to interact with foods by releasing active components with biological properties (e.g., antioxidant and antimicrobial capacity), which may be obtained from natural sources, such as plants, that are in line with the increased demand by consumers for food products with natural additives over synthetic ones [15]. Other active packaging systems include the use of exudate absorbent pads placed underneath the product, in order to avoid product deterioration due its contact with moisture. These pads have been used in packages of meat, fish and small soft fruits such as fresh strawberries [16]. Although biodegradable polymers (e.g., cellulose and its derivatives, polyvinyl alcohol and starch) have been used to produce such pads [17], there is still a need to test novel materials for this application. Chitosan, a polysaccharide produced from various fungi and the shell waste of shrimps, crabs and bones of cuttlefish and squids, is an example. It is non-toxic, with an allergen-free nature (approved by the FDA and European Union) and possesses antimicrobial and antioxidant activities [18,19,20]. In addition, it is an effective carrier of antioxidant and antimicrobial agents, namely those of aromatic plant extracts, such as green tea and rosemary [20,21,22]. Rosemary plant is a good source of bioactive compounds, including flavonoids, phenolics, diterpenoids and triterpenes [23,24]. Extracts from rosemary have shown strong inhibitory activity against various bacteria strains [25], and high antifungal activity [26,27]. Furthermore, the green tea plant, rich in polyphenols and catechins, is also associated with health benefits and antimicrobial and antioxidant properties [28]. Previous research studies proved that the application of a chitosan film or coating incorporating natural antioxidant and antimicrobial agents improves the storability of several perishable foods [29,30,31,32,33,34].

Recently, novel chitosan active film-pads containing antifungal plant extracts (from green tea and rosemary) were developed. They have shown simultaneously great performance in terms of antimicrobial capacity against *P. expansum* and water absorption capacity, without compromising their mechanical resistance under high moisture conditions [35]. As such, chitosan may be used to produce dense hydrophilic films with added active plant extracts with interesting water absorption, showing potential to be used as fruit pads.

Fresh berries are highly susceptible to spoilage during storage due to microbial growth. To the best of our knowledge, there are no reported studies regarding the application of chitosan films enriched with aromatic plant extracts as active pads in the preservation of soft fruits. As such, this study is focused on the application of packaging with increased sustainability, composed of chitosan active film-pads with antifungal plant extracts (green tea and rosemary) inside a commercial tray sealed with a compostable polymer (PLA) film, aiming at the extension of fresh red raspberry fruits’ shelf life.

## 2. Materials and Methods

### 2.1. Materials

To produce active film-pads, we used chitosan powder (Golden/Shell Biochemical Co., Ltd, Zhejiang, China), lactic acid (Panreac Quimica SAU, Barcelona, Spain), glycerol (Fisher Scientific, Loughborough, UK), dried leaves of commercial green tea (*Camellia sinensis* (L.) Kuntze) from Azores Island (Gorreana, São Miguel, Portugal) and rosemary (*Rosmarinus officinalis* L.), and ethanol (96% vol.) (Valente e Ribeiro. Lda, Lisbon, Portugal). For fruit preservation studies, we used red raspberries (*Rubus idaeus* L., cv. ‘Kweli’) acquired from a local farm in the Beja district, Portugal (37.65423, −8.756739), and a polylactic acid (PLA) compostable film (VGB 4 Vegware, London, UK) (O_2_ permeability: 2.97 × 10^−16^ m^2^ s^−1^ and CO_2_ permeability: 1.22 × 10^−15^ m^2^ s^−1^). In the analytical methods, we used analytical grade sodium hydroxide (Merck Life Science S.L.U., Algés, Portugal), methanol, HCl, gallic acid, 1,1–diphenyl-2-picrylhydrazyl, Trolox, 2,4,6-Tris(2-pyridyl)-s-triazine (TPTZ) and FeCl_3_·6H_2_0 from Panreac Quimica SAU, Barcelona, Spain.

### 2.2. Development of the Active Film-Pads

Chitosan powder was dissolved in a lactic acid solution (1% *v*/*v*) at a concentration of 2% *w*/*v*. Glycerol was added as a plasticizer at a concentration of 25% *w*/*w* (chitosan basis). The resulting solution was transferred to flat plastic containers (0.4 mL cm^−2^) and then dried at a temperature of 45 °C and 30% relative humidity for 24 h. After drying, the chitosan-based film obtained was cut into rectangular pads (7 cm × 5 cm).

Ethanolic extracts were obtained according to Vieira et al. [35]. Two different plants were used: green tea (*Camellia sinensis* (L.) Kuntze) and rosemary (*Rosmarinus officinalis* L.), both purchased from a local market in Lisbon (Portugal). The commercial dried plant leaves were stored at 5 °C in the dark until use. For each extract, the plant material was mixed with ethanol (96% vol.) in a 1:10 ratio (*w*/*v*). After vigorous stirring for 30 min protected from light at room temperature, the mixture was filtered using a Whatman No. 1 filter paper and a vacuum pump (Yuchengtech, AP-9925, Beijing, China ). To avoid degradation, the ethanolic extracts of green tea (GTE) and rosemary (RSME) were stored in the dark at 4 °C for further use.

The active film-pads were then prepared by immersion of the rectangular pads into GTE and RSME ethanolic extracts in sealed Petri dishes, overnight at 4 °C, followed by drying at room temperature protected from light. The average pad thickness was around 215 µm, measured using a digital micrometer (APB-3D Mitutoyo, Kanagawa, Japan) [35].

### 2.3. Raspberry Fruits Preservation Using Packages with Active Antifungal Film-Pads

The fresh red raspberries (*Rubus idaeus* L., cv. ‘Kweli’) were acquired at commercial maturity. Fruits were selected to be free from visible defects and injuries, to avoid interference from natural infections before any packaging treatment.

The antifungal active film-pads were placed on the bottom of commercial packages for red raspberries. Packages were trays, which were wrapped with a polylactic acid (PLA) compostable film and heat-sealed, under normal atmospheric conditions, to promote passive MAP conditions [1]. A total of 80 sealed trays were stored at 4 °C and 95% of relative humidity divided into four groups: trays without film-pads (Ctr); trays with chitosan film-pads but without extracts incorporated (Ch); trays with chitosan film-pads containing green tea extract (Ch + GTE); trays with chitosan film-pads containing rosemary extract (Ch + RSME).

The fruits were analyzed over time (at 0, 3, 7, 14 days of storage at 4 °C), in terms of surface color, weight loss, firmness, pH, total soluble solids, volatile acidity, total phenolic content and antioxidant activity. The composition of the atmosphere inside fruit packages was also measured over time. Five independent packages were analyzed for each group on each day.

### 2.4. Analytical Methods

#### 2.4.1. Composition of the Atmosphere inside the Packages

The concentration of carbon dioxide (CO_2_) and oxygen (O_2_) inside packages was measured using a gas analyzer (Checkmate 9900, PBI Dansensor, Ringsted, Denmark). An adhesive silicon septum was glued to the sampling point of packages to prevent gas leakage during analysis. The needle of the gas analyzer was inserted through the septum and results are expressed in percentages of O_2_ and CO_2_. Five packages per group were analyzed on each test day.

#### 2.4.2. Fungal Decay Incidence

Fungal decay was visually inspected after 3, 7 and 14 days of storage at 4 °C. Raspberry fruit showing surface mycelia development was considered decayed. Fungal decay incidence was quantified as the percentage of total fruits that showed surface mycelia development.

#### 2.4.3. Weight Loss, Firmness and Surface Color

The weight loss (% from the original weight [36]) from each sealed tray was measured using an electronic balance (TC-403, Denver Instrument Company, Vernon Hills, IL, USA). The results are expressed as an average of five replicates per group for each test day.

A Texturometer (TA-XT2, Stable Micro System, Surrey, UK) with a 5 kg load cell and equipped with a flat probe (37 mm diameter) was used to evaluate firmness in whole fresh raspberries. A number of 15 fruits from each package (three packages, randomly chosen from the total five packages used per group and test day) was assayed. Each fruit was positioned under the probe and compressed to 80% deformation at a speed of 2 mm s^−1^. All fruit samples were tempered at least for 6 h at 25 °C before measurements.

A Konica Minolta CTR-300 colorimeter (Minolta, Williams Drive Ramsey, NJ, USA) was used to measure L* (lightness), a* (red-green) and b* (yellow-blue) color parameters of raspberries. It was calibrated with a standard white plate, which was provided by the manufacturer. The color was measured on the non-moldy fruits from each package (three packages, randomly chosen from the total five packages used per group and test day). Measurements were taken on the side of a slightly flattened whole fruit [36].

#### 2.4.4. Total Soluble Solids Content, pH and Volatile Acidity

The total soluble solids (TSS), pH and volatile acidity were measured in a fruit pulp produced by trituration of 15 fruits selected per tray (three packages, randomly chosen from each group per test day), using an Ultra-Turrax blender (IKA T18 basic Ultra-Turrax, Staufen, Germany).

The determination of TSS was performed using the clear juice obtained by pulp filtration with a double layer of gauze. The TSS was measured with a refractometer (Atago, Fisher Scientific, Ga., Bellevue, WA, USA). The pH values were measured using by a pH meter (Russel, Moder RL) with the electrode being directly immersed in the pulp [37].

The measurement of volatile acidity was carried as described in ISO 6632-1981 [38] with minor modifications. A mixture of the fruit pulp (10.0 g) and deionized water (10 mL) was steam-distilled using an automatic distiller (Gerhardt, Königswinter, Germany). The distillate was collected (250 mL), and the volatile compounds were neutralized with 0.1 M sodium hydroxide (Eka Pellets™, Amsterdam, The Netherlands) in the presence of 1% phenolphthalein (Himedialabs, Esdoornlaan, DB Maarn, The Netherlands) as an indicator. The consumption of 0.1 M sodium hydroxide represented the volatile acidity, being expressed in mass of acetic acid per mass of raspberries (mg acetic acid 100 g^−1^).

#### 2.4.5. Total Phenolic Content and Antioxidant Activity

For the quantification of total phenolic content and the antioxidant activity of packed raspberry fruits, extracts were obtained using a modified method from Kopjar et al. [39]. Five grams of raspberry pulp was extracted with 15 mL of methanol/HCl (99/1, *v*/*v*). The mixture of pulp and solvent was well mixed using a magnetic stirrer in the dark at 10 °C for 1 h. Then, the mixture was centrifuged at 14,881× *g* for 10 min at 4 °C and the supernatant was recovered, obtaining a final volume of 50 mL. The extraction was repeated three times for each group and test day. The supernatant was maintained at 4 ºC in the dark before analytical determinations.

##### Total Phenolic Content (TPC)

TPC was analyzed by measuring the absorbance of the prepared extracts at a wavelength of 280 nm (UNICAM, UV/Vis spectrometer-UV4, Waltham, MA, USA), as described in other works in the literature [40,41]. Gallic acid (St. Louis, MO, EUA) was used as standard, and a calibration curve was performed measuring the absorbance of different gallic acid aqueous solutions (from 0 mg L^−1^ to 50 mg L^−1^). The TPC was expressed as gallic acid equivalents (GAE) per mass of raspberry fresh weight (mg GAE 100 g^−1^).

##### Antioxidant Activity (AOA)

1,1–diphenyl-2-picrylhydrazyl (DPPH) Assay

The DPPH method was used as described by Huang et al. [42], with minor modifications. A mass of 24 mg of DPPH was dissolved in 100 mL of methanol to make a stock solution. Before using, this stock solution was stored in the freezer for more than 2 h. A working solution was prepared by mixing 10 mL of stock solution with 45 mL of methanol to achieve an absorbance of less than 1.1 at 515 nm. An aliquot of 0.1 mL of extract was added to 4.9 mL of DPPH working solution and the absorbance was measured after 40 min (*Abs sample*). The analysis was carried out in triplicate. The blank consisted of 4.9 mL of DPPH working solution with 0.1 mL of methanol (*Abs blank*). The RSA (radical scavenging activity) was calculated by Equation (1).

(1) $$RSA\left(\%\right)=\frac{Abs\;blank-Abs\;sample}{Abs\;blank}\times 100$$

Trolox was used as standard, and a calibration curve was performed correlating RSA values with different Trolox concentrations (from 100–2000 µM). The results were expressed as Trolox Equivalent Antioxidant Capacity (TEAC), as mmol Trolox per mass of fresh raspberry (mmol Trolox 100 g^−1^).

FRAP Assay

The FRAP test was performed using a modified version of that used by Suárez et al. [43]. A volume of 25 mL of acetate buffer 0.3 M was mixed with 2.5 mL of 2,4,6-Tris(2-pyridyl)-s-triazine (TPTZ) 0.01 M and 2.5 mL of Iron(III) chloride hexahydrate (FeCl_3_·6H_2_O) solution 0.02 M to produce the working FRAP mixture. An aliquot of 90 µL of extract was added to a mixture of 270 µL of deionized water and 2.7 mL of the working FRAP solution. The absorbance was measured at 595 nm after reaction in a water bath (37 °C) for 40 min. The analysis was carried out in triplicate. Trolox was used as standard, and a calibration curve was performed correlating the absorbance at 595 nm with different Trolox concentrations (from 100–2000 µM). The results were expressed as Trolox Equivalent Antioxidant Capacity (TEAC), as mmol Trolox per mass of fresh raspberry (mmol Trolox 100 g^−1^).

### 2.5. Statistical Analysis

After checking the normal distribution, ANOVA and Tukey tests were used to assess if there were significant differences between the data’s average values, for a significance level of 0.05. Data analysis was performed using the software STATISTICA TM version 8.0 (StatSoft Inc., Tulsa, OK, USA).

## 3. Results and Discussion

### 3.1. Composition of the Atmosphere inside the Packages

**Figure 1:**
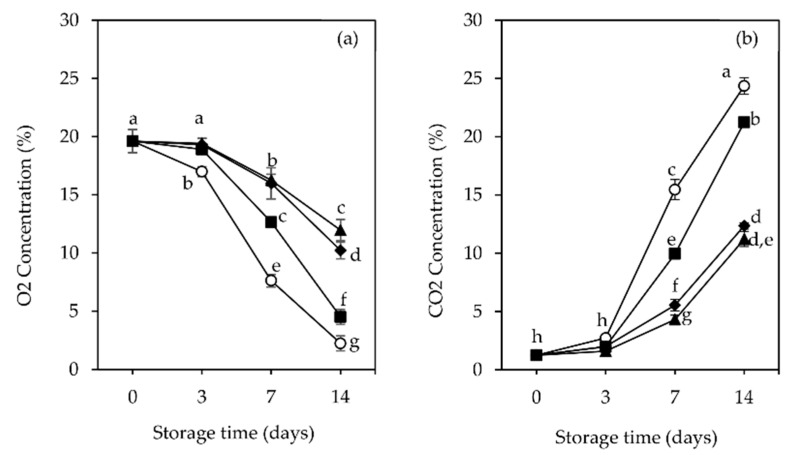
Changes in headspace gas composition: (**a**) O_2_ and (**b**) CO_2_ concentrations (%), of raspberry (*Rubus idaeus* L. cv. ‘Kweli’) fruit packages (no film-pad_Ctr, ○; with film-pad containing green tea extract_Ch + GTE, ◆, rosemary extract_Ch + RSME ▲, only chitosan ■), stored for 14 days at 4 °C under passive MAP conditions. Vertical bars indicate standard deviation of five replicates. Means with equal letters are not statistically different by Tukey’s test with 5% significance level.

The O_2_ and CO_2_ contents detected inside the packages for raspberries are reported in Figure 1.

The initial atmosphere gas composition (19% O_2_ and 1.25% CO_2_) changed in the packages across all the packaging treatments applied. The exchange area (310.5 cm^2^) through the film packages was constant, so the evolution of the atmosphere inside the trays was passively created by the respiration rate of the fruits, the metabolic activity of fungal development and the permeability of the film to O_2_ and CO_2_ [44], which are affected by temperature [1]. Nevertheless, significant differences (*p* < 0.05) were observed when comparing raspberry fruits in the presence of different film-pads.

The O_2_ content in control and in Ch samples (both treatments without the extracts incorporated) were significantly much lower (*p* < 0.05) (Figure 1a) during storage, achieving minimal levels around to 2.24% and 4.52%, respectively, at the end of 14 days of storage, compared to the levels reached by the active film-pad samples Ch + GTE (10.91%) and Ch + RSME (11.95%). Given that the raspberries are non-climacteric, the drop in O_2_ content may be related to O_2_ consumption due to metabolic activity of fungal development [45]. Indeed, the control and Ch samples presented higher fungal incidence and decay compared to Ch + GTE and Ch + RSME samples (Section 3.2), which were significantly (*p* < 0.05) much lower. In these latter groups, possible interactions between antifungal agents from GTE and RSME loaded on chitosan and the fruit surface existed, along with pads’ high water absorption capacity [35], which could absorb and inhibit fungal droplet moisture.

CO_2_ contents were low in the first 3 days of refrigerated storage, without significant differences (*p* > 0.05) between all package groups (Figure 1b). After the third day, the content increased quickly, and at seventh day, it increased with significant differences (*p* < 0.05) between package groups. At the end of 14 days of storage, the CO_2_ content in control samples packages reached the highest values (24.36%), followed by those with Ch pads (21.24%), which were significantly (*p* < 0.05) different, compared to the lowest CO_2_ levels reached with Ch + GTE (12.35%) and Ch + RSME pads (11.21%). At this point, the high CO_2_ atmosphere and minimal O_2_ levels reached in package samples without any antifungal active film-pads inside became potentially harmful to fruit quality. According to Dennis et al. [46], the induction of anaerobic respiration can cause multiple undesirable changes in the fruits, including the development of off-flavors [47]. On the other hand, the O_2_ and CO_2_ levels in package samples containing Ch + GTE and Ch + RSME pads were around 10% and 10–20%, which are recommended as desirable to preserve fresh raspberry quality [48]. Indeed, the fruit quality in the presence of the antifungal active film-pads enhanced for 14 days of storage at low temperature. In contrast, fruits in package samples that did not contain the active film-pads became not marketable by visual analysis (Section 3.2).

### 3.2. Fungal Decay of the Raspberry Fruits

**Figure 2:**
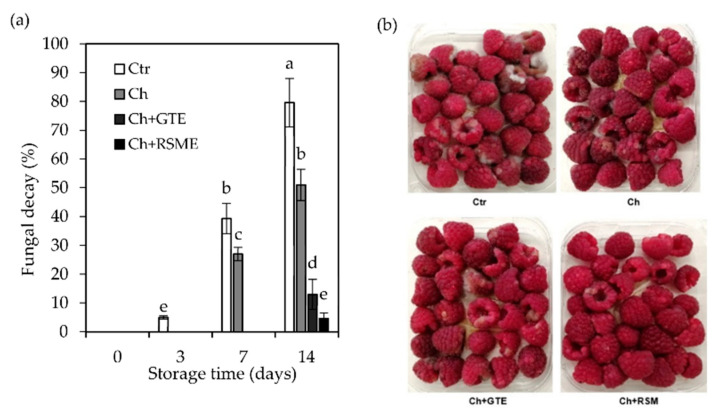
Evolution of fungal decay (**a**) and appearance at 14 days of storage (**b**) of packaged raspberry (*Rubus idaeus* L. cv. ‘Kweli’) fruits (no film-pad _Ctr; with film-pad containing green tea extract_Ch + GTE, rosemary extract_Ch + RSME and only chitosan_Ch) stored for 14 days at 4 °C under passive MAP conditions. Means with equal letters are not statistically different by Tukey’s test with 5% significance level.

Raspberry fruit is highly perishable, due to high susceptibility to mechanical injury, water loss, high metabolic activity and mold and rot growing. To determine the effectiveness of the antimicrobial active film-pads for enhancing the fruit quality, fungal decay as the primary determinant of quality was observed during the storage time and statistically compared to the control samples (Figure 2).

Fungal decay started quickly on the control samples, with 5% of fruits displaying signs of infection at day 3 of storage, and 39.4% displaying signs of infection at 7 days of storage at 4 °C. At the end of storage, we recorded 79.5% damage by mold spoilage in fruits in control samples, which was significantly higher (*p* < 0.05) than that of the other package groups (packages with film-pads) (Figure 2a). At this point, most of the raspberries in control samples showed the development of fungus *Botrytis cinerea* ‘gray mold’ mycelium and slightly brown spots (Figure 2b). The water vapor transmission rate of the PLA film used to wrap the trays is low regarding the transpiration rate of fresh raspberry fruits [49]. Therefore, high relative humidity conditions prevailed in the packages, causing condensation of water vapor and enhancing the conditions for fungal growth (in the absence of antimicrobial agents), resulting in fruit spoilage. In the case of Ch packages, the first signs of decay started to appear at day 7, showing 12.35% less infection than control packages, and at the end of 14 days, differences were significantly much higher (*p* < 0.05), showing 28.54% less decay. Chitosan was previously reported to have an antifungal effect when applied on raspberries [50], as well as on other soft fruits such as strawberries under cold storage [51,52]. Nevertheless, when fruits were packaged with the antifungal active film-pads (Ch + GTE and Ch + RSME), the fungal incidence and decay were observed only at the end of 14 days of storage, and differences were significantly much higher (*p* < 0.05) than control samples, with 66.54% and 74.92% less fungal decay, respectively. The Ch + RSME film-pad significantly (*p* < 0.05) reduced decay in raspberry fruits to less than 5% of spoiled fruits, compared to 13% with Ch + GTE active film-pad and 51% with Ch film-pad. The release of phenolic compounds from active film-pads to raspberries upon direct contact between them limits fungal growth. Furthermore, as some of the compounds of the extracts are volatile, their release and accumulation in the internal package atmosphere may also have played an important role in the antifungal activity. The suppressed spoilage decay over 14 days with Ch + GTE and Ch + RSME pads compared to control samples may have important economic implications, as the shelf life of raspberry fruits can be extended in the fresh market. Other reports have shown that chitosan antifungal activity can be enhanced by the addition of bioactive compounds such as lemon essential oil when applied to cold storage of soft fruits [53,54].

### 3.3. Weight Loss, Firmness, and Surface Color

**Figure 3:**
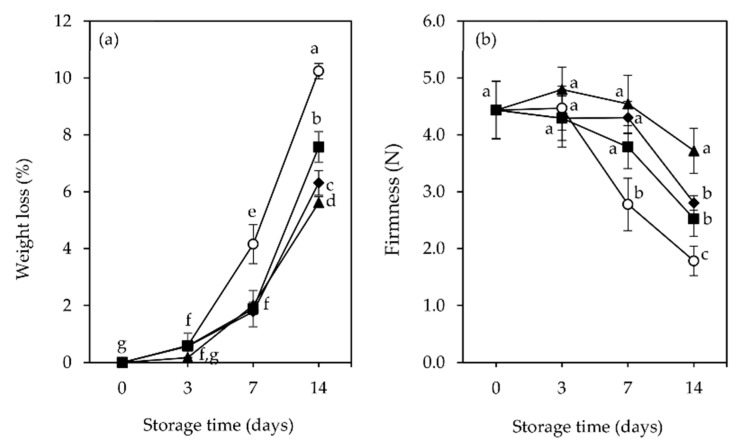
Changes in weight loss (**a**) and firmness (**b**) of packaged raspberry (*Rubus idaeus* L. cv. ‘Kweli’) fruits (no film-pad _Ctr, ○; with film-pad containing green tea extract_Ch + GTE, ◆, rosemary extract_Ch + RSME ▲, only chitosan ■), stored for 14 days at 4 °C under passive MAP conditions. Vertical bars indicate standard deviation of five replicates. Means with equal letters are not statistically different by Tukey’s test with 5% significance level.

As a result of their high water content (80–95%) and thin skin, raspberries present a high tendency to decrease in mass due to water loss. The effect of the active film-pads on raspberry fruits’ weight loss is depicted in Figure 3a.

The data indicate that the % loss across all treatments increased with increasing storage period. No significant differences (*p* > 0.05) between the different film-pads tested were observed during the 7 days of storage. On the other hand, the weight loss in control samples sharply increased after the third day of storage, distancing from all the film-pad samples during storage and reaching the highest weight loss (10.24%), followed by Ch film-pad (7.57%) at the end of 14 days of storage. In contrast, the fruits packaged with the active film-pads Ch + GTE and Ch + RSME had the lowest weight loss (6.31% and 5.61%, respectively), which was significantly different from the Ch samples. Thus, the study indicates that both active film-pads Ch + GTE and Ch + RSME, by repressing fungal growth inside raspberry packages under cold storage (Section 3.2), retarded fruit tissue deterioration, which simultaneously contributed to reduced fruit water loss, along with changes in the respiration process (Section 3.1), the main metabolic process that is linked to moisture loss [50].

Firmness maintenance is one of the most important physical attributes for the quality of raspberries [50]. Significant differences (*p* < 0.05) between the film-pad samples and the control samples were observed in the loss of firmness during storage (Figure 3b). The firmness of control samples rapidly decreases after day 3, with a significant (*p* < 0.05) loss in firmness of 61% at day 14 in relation to the initial fruit firmness at day 0. The fruits packaged with Ch and Ch + GTE film-pads remained firmer over 7 days of storage, but after this period, both treatments were not effective, and fruits suffered an average loss in firmness at day 14 of 44.4% and 38.8%, respectively. Contrarily, when Ch + RSME was applied inside packages, the fruits remained much firmer during the storage period, with a lower loss in firmness (16.7%), significantly (*p* < 0.05) lower compared to that of fruits of the other packages at the end of 14 days. The differences in fruit firmness between type of packages may be related to moisture loss [55]. Indeed, raspberries packaged with Ch + RSME pads showed the lowest average weight loss rate during the storage period (Figure 3a), contributing to the maintenance of fruits’ tissue integrity.

The chromatic characteristics of raspberries packaged with the film-pads as the function of storage time are shown in Table 1.

The CIELab color space was used, measuring three coordinates: L*, the lightness, as well as a* and b*, which are the green-red and blue-yellow color components, respectively. The a*/b* ratio has been used as a color index in various types of fresh fruits [56]. The raspberry fruits before treatments presented a bright red color, and the initial L* and a*/b* ratio values were 27.37 and 2.17, respectively. Furthermore, the external color derived from anthocyanins is related to consumers’ perception of quality and is an important parameter for visual ripeness and freshness assessments of raspberry [57]. Statistical analysis showed that the interaction of package type and storage time was significant for L* and a*/b* ratio parameters. L* values were constant with no significant differences (*p* > 0.05) for all package types during the first three days of storage. After that, differences gradually became more significant (*p* < 0.05), as raspberry fruits in control packages and in packages with Ch pads lost more of their luminosity (showed greater decreases in L*) than berries in Ch + GTE and Ch + RSME packages, with L* values of 26.56 and 26.28, respectively, at the end of storage. A decrease in the L* value reflected the darkening of fruits by anthocyanin accumulation and indicated that the ripening process had occurred in the fruits [57]. Contrarily to Briano et al. [1], we found no relation between the CO_2_ accumulation inside packages in control samples and its effect on the maintenance of L* value. Furthermore, the raspberries in Ch + RSME and Ch + GTE packages showed the highest L* value (27.10 and 26.75) at the end of storage. According to Perdones et al. [54] and Duran et al. [58], high moisture loss can be linked to the darkness on fruits. Indeed, raspberries from Ch + RSME and Ch + GTE packages showed the lowest weight loss during storage (Figure 3a). During storage, the color of fruits in control and Ch packages became less reddish on the seventh day, suggesting the degradation of anthocyanins during the ripening process and browning reactions that are typical of fruit senescence [57], and this trend was significantly (*p* < 0.05) more evident at the end of 14 days of storage. Indeed, fruits in control in Ch packages showed the lowest a*/b* ratio values of 2.01 and 2.05, respectively, typified by a red-yellow color and a lower aesthetic appeal enhanced by fungal spoilage. In contrast, Ch + RSME and Ch + GTE samples showed better color retention, with a more vivid red color, given the higher a*/b* ratio values of 2.24 and 2.20, respectively, at the end of 14 days of storage, which were not significantly (*p* > 0.05) different from the initial value observed at day 0.

### 3.4. Total Soluble Content, pH and Volatile Acidity

Total soluble solids (TSS) content is an important parameter that affects fruit quality and consumer acceptability [59], and high values are required for good berry flavor [60]. The results obtained from the TSS analysis are presented in Table 2. TSS content in raspberry fruits decreased gradually during storage time, and significant differences were observed when comparing different package types. The TSS values of fruits packaged with Ch + RSME pads slightly decreased from 9.53% at time 0 to 9.35% at day 3. The values remained at 9.33 through 4 days followed by a decline to 8.53% at the end of storage. Regarding the TSS values of fruits in Ch + GTE and Ch packages, although presenting the same decreasing behavior of those packaged with Ch + RSME pads during storage time, the values were significantly lower (*p* < 0.05), reaching values of 7.95% and 7.58% for packages with Ch + GTE and Ch pads, respectively, at the end of storage. In addition, the TSS content in control samples dropped sharply during storage and was significantly different (*p* < 0.05) from the values of fruits packaged with Ch + RSME and Ch + GTE pads, achieving the lowest value of 7.43% at 14 days of storage. This decrease in TSS can be explained by the fact that the hydrolysis of sucrose to maintain physiological activity was more rapid [59]. Indeed, the results showed that fruits from the control packages had enhanced fungal growth and decay (Section 3.2) and presented the highest respiration rate (Section 3.1), as well as the highest weight loss by transpiration (Section 3.3), during the storage period, in comparison with the fruits packaged with active film-pads (particularly with the Ch + RSME).

Changes in pH values of raspberries after packaged with different film-pads during cold storage are also shown in Table 2. This parameter, which is related to the fruit senescence [61], increased significantly during storage due to the utilization of organic acids during respiration [62]. The pH difference between day 7 and day 14 was not statistically significant (*p* > 0.05) for all package types. However, pH values of fruits of control and Ch packages were significantly (*p* < 0.05) higher than those of fruits packaged with Ch + GTE and Ch + RSME pads at day 7 and day 14, indicating that chitosan combined with green tea and rosemary extracts can delay changes in the pH of raspberry fruits.

Volatile acidity (VA) is another important parameter related to sensory appreciation. High VA values indicate the accumulation of acetic acid, which gives an off-flavor, such as a vinegar or acidic flavor, as result from redox reactions of ethanol and acetaldehyde [63], which are both by-products of the fermentative metabolism in over-ripe and senescent fruits [64]. The VA values of raspberry fruits increased significantly (*p* < 0.05) during the refrigerated storage for all package types (Table 2). Indeed, the VA values in control and Ch packages rapidly increased after day 3 and day 7, achieving the highest values of 0.415 and 0.235, respectively, at the end of storage. In contrast, the VA values for fruits packaged with Ch + GTE and Ch + RSME pads had a slower increase after day 3 and day 7, achieving the lowest values of 0.150 and 0.105, respectively.

### 3.5. Total Phenolic Content and Antioxidant Activity

**Figure 4:**
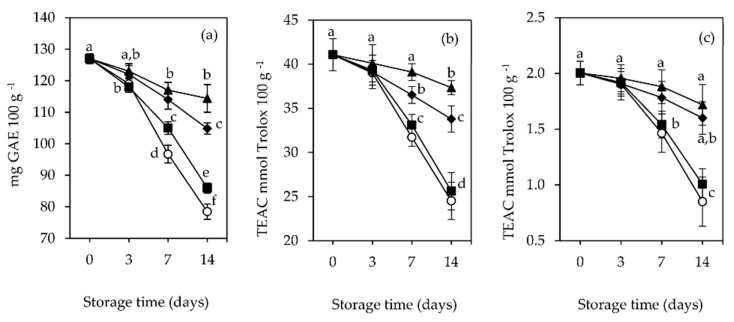
Total phenolic content (**a**) and antioxidant activity (AOA) [by DPPH (**b**) and FRAP (**c**) methods] of packaged raspberry (*Rubus idaeus* L. cv. ‘Kweli’) fruits (no film-pad_Ctr ○; with film-pad containing green tea extract_Ch + GTE ◆, rosemary extract_Ch + RSME ▲, only chitosan ■), stored for 14 days at 4 °C under passive MAP conditions. Vertical bars indicate standard deviation of three replicates. TEAC: Trolox equivalent antioxidant capacity; GAE: gallic acid equivalents. Means with equal letters are not statistically different by Tukey’s test with 5% significance level.

The initial total phenolic content (TPC) of raspberry fruits was 127 mg GAE 100 g^−1^, and the respective antioxidant activity (AOA) was 41.4 mmol Trolox 100 g^−1^ with the DPPH method and 2.0 mmol Trolox 100 g^−1^ with the FRAP method. Figure 4 represents the changes in TPC and AOA of raspberries over the storage time at 4 °C, packaged with the different film-pads.

As shown in Figure 4a, the TPC of raspberry fruits varied over time for all types of packages used. The maximum retention of TPC was found for fruits packaged with Ch + RSME pads (90.1%), followed by those of Ch + GTE packages (82.5%), at the end of the 14th day. The TPC of fruits of control and Ch packages showed a sharp decline after the third day of storage and the highest decrease (of 61.8% and 67.7%, respectively) at the end of storage. At 7 and 14 days of storage, the retention of TPC of fruits packaged with Ch + RSME and Ch + GTE pads was significantly (*p* < 0.05) different compared to TPC of fruits from control and Ch packages. Yang et al. [65] found a link between TPC values and the amount of several compounds, such as catechin and epicatechin, as well as phenolic acids (e.g., gallic and caffeic acid), reported to be present in raspberries. Ponder and Halmman [66] also reported the presence of myricetin, luteolin and kaempferol in these fruits. The rapid decline in the TPC values in fruits from control and Ch packages may be attributed to its higher respiration rate, resulting in the breakdown of total phenols [67], which may be related to the high fungal incidence, beginning on the third day for control packages and on the seventh day for Ch packages (Section 3.2), which progressively increased thereafter, causing decay in the majority of fruits (79.5% and 51%, respectively) at the end of storage, given the absence of the antifungal GTE and RSME in both cases.

The results of this work reveal a considerable variation in the AOA of raspberry across the different packaging types. In the DPPH and FRAP assays, a similar trend was observed for fruits from all the package types (Figure 4b,c). The highest AOA obtained with DPPH and FRAP methods at the end of the storage period was recorded for fruits from Ch + RSME packages (37.3 and 1.7 TEAC mmol Trolox 100 g^−1^, respectively), followed by fruits from Ch + GTE packages (33.8 and 1.6 TEAC mmol Trolox 100 g^−1^). On the other hand, the lowest AOA was observed for fruits from control (24.5 and 0.9 TEAC mmol Trolox 100 g^−1^, respectively) and Ch packages (25.6 and 1.01 TEAC mmol Trolox 100 g^−1^, respectively). As such, the antioxidant potential was found to be comparatively higher for the fruits packaged with the active film-pads. From those, the Ch + RSME film-pad was more effective in retaining the fruits’ AOA during storage. The presence of the active film-pads inside raspberry fruit packages, by inhibiting fungal growth and spoilage (Section 3.2), seems to enhance the effect of packaging conditions applied, slowing down the metabolism in fruits during storage (Section 3.1 and Section 3.3), thus minimizing the metabolism consumption of phenolics and flavonoids [68]. Under storage, secondary metabolites such as phenolics accumulate, causing an increase in the antioxidant levels [69].

**Figure 5:**
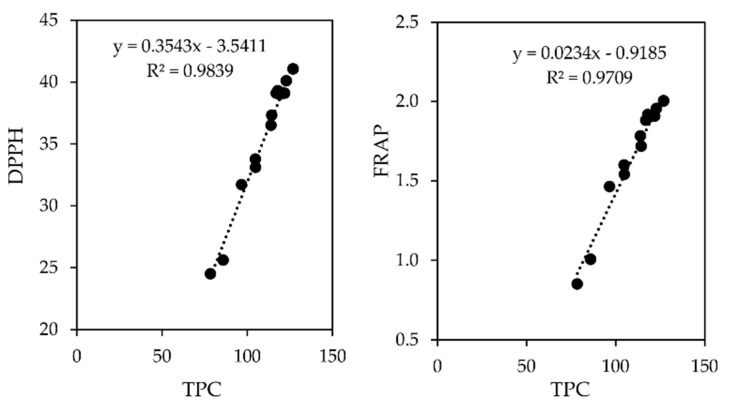
Correlations between AOA measured by DPPH and FRAP methods (TEAC mmol Trolox 100 g^−1^) with TPC (mg GAE 100 g^−^^1^). For both correlations, all data of the study (all sample types and all days of storage) were used.

Significant positive correlations (*p* < 0.05) were observed between TPC and AOA by DPPH and FRAP methods (Figure 5).

The strong correlations observed indicate that the AOA of raspberries is mainly due to their composition in phenolic compounds. The different TEAC values obtained for the two methods are attributed to the different types of reactions taking place. The DPPH approach relies on radical scavenging, whereas the in the FRAP method, there is a reduction of Fe^3+^ [70]. These findings are consistent with those previously published [71], indicating that the FRAP and DPPH procedures are suitable for assessing antioxidant activity in raspberry fruit extracts with suitable reproducibility.

## 4. Conclusions

In this work, packaging strategies composed of trays sealed with compostable polylactic acid films and containing active film-pads made of chitosan with the incorporation of green tea (Ch + GTE) and rosemary ethanolic extracts (Ch + RSME) were tested in post-harvest preservation of raspberry fruits. The antifungal active film-pads tested in this work successfully decreased raspberry fruit fungal incidence, maintaining overall quality, as reflected in the fruits’ physicochemical properties during storage, thereby extending the fruits’ shelf life up to 14 days. These results can be attributed to the active compounds extracted from green tea and rosemary, acting as natural antifungal agents. At the end of storage (day 14), raspberry fruits packaged with both active film-pads maintained the most important qualitative and nutraceutical traits, close to those at harvest time, in comparison with fruits packaged without film-pads. Still, fruits packaged with Ch + RSME pads could be recommended, taking into account all the physical, chemical and microbial parameters studied. This green active packaging strategy may be suitable for other soft fruits to increase their marketability and as an effective substitute for non-biodegradable polymeric materials, applicable for products with clean and natural labels.

## Figures and Tables

**Table 1:** Changes in color values (L* and a*/b*) of packaged raspberry (*Rubus idaeus* L. cv. ‘Kweli’) fruits (no film-pad _Ctr; with film-pad containing green tea extract_Ch + GTE, rosemary extract_Ch + RSME and only chitosan_Ch) stored for 14 days at 4 °C under passive MAP conditions.

Type of Package	Storage Time (Days)			
	0	3	7	14
	L* values			
Ctr	27.37 ± 0.47 ^abA^	27.47 ± 0.31 ^aA^	26.54 ± 0.29 ^bB^	26.28 ± 0.19 ^bcAB^
Ch	27.37 ± 0.47 ^aA^	27.44 ± 0.24 ^aA^	26.89 ± 0.24 ^aAB^	26.56 ± 0.18 ^aAB^
Ch-GTE	27.37 ± 0.47 ^aA^	27.36 ± 0.41 ^aA^	27.23 ± 0.28 ^aA^	26.75 ± 0.22 ^aA^
Ch-RSME	27.37 ± 0.47 ^aA^	27.56 ± 0.28 ^aA^	27.24 ± 0.24 ^aA^	27.10 ± 0.29 ^aA^
	a*/b* values			
Ctr	2.17 ± 0.04 ^aA^	2.24 ± 0.04 ^aA^	2.13 ± 0.08 ^abA^	2.01 ± 0.07 ^bB^
Ch	2.17 ± 0.04 ^aA^	2.21 ± 0.06 ^aA^	2.16 ± 0.05 ^abA^	2.05 ± 0.06 ^bB^
Ch-GTE	2.17 ± 0.04 ^aA^	2.19 ± 0.05 ^aA^	2.22 ± 0.08 ^aA^	2.20 ± 0.07 ^aA^
Ch-RSME	2.17 ± 0.04 ^aA^	2.23 ± 0.014 ^aA^	2.20 ± 0.05 ^aA^	2.24 ± 0.02 ^aA^

Data are means ± SD of three replicates. Means in each row with the same letters (a–c) and means in each column with the same letters (A,B) are not significantly different (*p* ≤ 0.05), according to Tukey’s test.

**Table 2:** Changes in TSS, pH and volatile acidity values of packaged raspberry (*Rubus idaeus* L. cv. ‘Kweli’) fruits (no film-pad _Ctr; with film-pad containing green tea extract_Ch + GTE, rosemary extract_Ch + RSME and only chitosan_Ch) stored for 14 days at 4 °C under passive MAP conditions.

Treatments	Storage Time (Days)			
	0	3	7	14
	*TSS values (%)*			
Ctr	9.53 ± 0.11 ^aA^	8.90 ± 0.24 ^bB^	8.48 ± 0.16 ^bC^	7.43 ± 0.15 ^cC^
Ch	9.53 ± 0.11 ^aA^	8.78 ± 0.14 ^bB^	8.76 ± 0.13b ^BC^	7.58 ± 0.16 ^cC^
Ch-GTE	9.53 ± 0.11 ^aA^	9.00 ± 0.14 ^bB^	8.87 ± 0.12 ^bB^	7.95 ± 0.12 ^cB^
Ch-RSME	9.53 ± 0.11 ^aA^	9.35 ± 0.07 ^aA^	9.33 ± 0.06 ^aA^	8.53 ± 0.06 ^bA^
	*pH values*			
Ctr	2.87 ± 0.01 ^cA^	2.96 ± 0.01 ^bA^	3.13 ± 0.02 ^aA^	3.17 ± 0.02 ^aA^
Ch	2.87 ± 0.01 ^cA^	2.94 ± 0.02 ^bA^	3.14 ± 0.02 ^aA^	3.16 ± 0.04 ^aA^
Ch-GTE	2.87 ± 0.01 ^cA^	2.92 ± 0.03 ^bA^	3.05 ± 0.01 ^aB^	3.10 ± 0.02 ^aB^
Ch-RSME	2.87 ± 0.01 ^bA^	2.88 ± 0.01 ^bB^	3.05 ± 0.01 ^aB^	3.07 ± 0.01 ^aB^
	*Volatile acidity values (mg acetic acid 100 g^−1^)*			
Ctr	0.059 ± 0.002 ^dA^	0.074 ± 0.037 ^cA^	0.150 ± 0.042 ^bA^	0.415 ± 0.021 ^aA^
Ch	0.059 ± 0.002 ^cA^	0.068 ± 0.025 ^cA^	0.120 ± 0.014 ^bAB^	0.235 ± 0.024 ^aB^
Ch-GTE	0.059 ± 0.002 ^bA^	0.065 ± 0.014 ^bA^	0.105 ± 0.021 ^aBC^	0.150 ± 0.019 ^aC^
Ch-RSME	0.059 ± 0.002 ^bA^	0.059 ± 0.015 ^bA^	0.075 ± 0.021 ^aC^	0.105 ± 0.021 ^aD^

Data are means ± SD of three replicates. Means in each row with the same letters (a–c) and means in each column with the same letters (A–D) are not significantly different (*p* ≤ 0.05), according to Tukey’s test.

## Data Availability

Data is contained within the article.
